# Effect of Artichoke Outer Bract Powder Addition on the Nutritional Profile of Gluten-Free Rusks

**DOI:** 10.3390/foods14132395

**Published:** 2025-07-07

**Authors:** Valentina Melini, Francesca Melini, Alessandro Salvati, Francesca Luziatelli, Maurizio Ruzzi

**Affiliations:** 1 CREA Research Centre for Food and Nutrition, Via Ardeatina 546, I-00178 Rome, Italy; francesca.melini@crea.gov.it; 2 Department for Innovation in Biological, Agro-Food and Forest Systems (DIBAF), University of Tuscia, I-01100 Viterbo, Italy; alessandro.salvati@studenti.unitus.it (A.S.); f.luziatelli@unitus.it (F.L.); ruzzi@unitus.it (M.R.)

**Keywords:** artichoke bracts, food waste, gluten-free, rusks, sustainability, phenolic compounds, dietary fiber, inulin

## Abstract

This study investigates the effect of incorporating outer bract powder on the bioactive compound content of gluten-free (GF) rusks, in terms of undigestible carbohydrates and phenolic compound content. The production of the artichoke powder as a functional ingredient was optimized by evaluating two key processing variables: drying time and pre-treatment of artichoke bracts with food-grade citric acid. Two distinct composite GF flour blends were used to formulate the GF rusks, and the nutritional quality thereof was systematically assessed. Results demonstrated that pre-treating the artichoke outer bracts with citric acid, followed by drying at 40 °C for 20 h, allowed for the production of a powder characterized by a lighter and reddish appearance, low fat content, and high dietary fiber level. The formulated rusks were rich in dietary fiber, whose intake is generally a deficiency in the diet of coeliac subjects. Furthermore, the enrichment with artichoke powder contributed to the production of a low-fat snack, in contrast with the GF snacks available on the market. The artichoke powder also showed a high content of free phenolic compounds, suggesting an enhanced dietary intake of antioxidants for consumers.

## 1. Introduction

Food by-products are secondary organic materials generated at various points along the food supply chain, such as farming, harvesting, post-harvesting, or food processing [1,2,3]. They are not intended for human consumption and are generally discarded and considered waste due to their unfavorable taste or texture [4]. Fruit and vegetable by-products (e.g., peels, skins, leaves, and stems) and grain and cereal by-products (e.g., bran, germ, and spent grain) are among the main categories of vegetable food by-products [4,5].

Disposal of these food biomasses represents a cost and negatively impacts the environment in terms of greenhouse gas emissions and landfill burden [5,6]. In order to support sustainable food systems and align food processing with the 12th Objective of the 17 development goals, laid down by the 2030 Agenda for Sustainable Development [7], new approaches have emerged in the management of food by-products. Leveraging food by-products through recycling and/or upcycling came out as a new frontier of investigation. It consents to valorize biomasses that are still rich in valuable compounds, such as dietary fiber, vitamins, minerals, sugars, and bioactive molecules, as well as to minimize waste, reduce environmental impact, and create new products and/or ingredients to be used in high-value applications [1,4,8,9].

Globe artichoke (*Cynara scolymus* L.) is part of the Italian culinary tradition and is appreciated for its flavor and taste. It is also praised for the nutritional quality: it is rich in vitamins and minerals, as well as in dietary fiber and phenolic compounds [10], which contribute to its antioxidant, hypocholesterolemic, and anti-inflammatory properties. The plant is known for the inulin content; this oligosaccharide has a low glycemic response, which makes it suitable for diabetics, and has a positive effect on the intestinal microbiota.

Among vegetable by-products, the residuals of artichoke processing represent an important waste biomass: 80–85% of artichoke production [11]. Stems and bracts are, in fact, discarded during harvesting and processing because the fibrous texture, potential bitterness, and/or lack of palatability make them unsuitable for consumption; in addition, residual leaves, stalks, and roots remain in the fields [11,12]. The amount of artichoke residual biomass, either disposed of as solid waste or left without any further valorization, can be huge in Italy, considering that the country is the main world producer of artichoke, with about four million tons produced yearly [13]. For this reason, it is important to investigate and identify alternative cycles for artichoke by-products.

Upcycling generally involves the transformation of agri-food residuals into powders or, less frequently, into extracts for incorporation into food categories such as bakery products, milk-based products, etc. Powders, flours, or extracts from agri-food by-products are used as functional ingredients in the formulation of bakery products, in order to improve their nutritional value, in terms of dietary fiber content and antioxidant compound content [1]. Among bakery products, rusks are crispy snacks that can be enjoyed at breakfast or tea-time, or in between meals. They have a longer shelf-life than regular bread, thanks to the low moisture content. They are a healthier option than bread substitutes. The nutritional profile of traditional rusks has been improved by the use of composite flours [14]. The application of composite flours or functional powder can contribute to addressing the nutritional deficiencies of coeliacs [15].

In this context, enriching GF rusks with artichoke powder could contribute to promoting a circular economy system and producing innovative and healthier products for subjects affected by coeliac disease.

Following literature analysis, it emerged that artichoke by-products were mostly upcycled into powder, upon freeze-drying [16,17,18,19,20,21,22], or were used as biomass to obtain extracts rich in phenolic compounds [23,24,25]. Oven-drying has been poorly applied to obtain powder from artichoke by-products [24,26,27]. Despite its low cost and easy application, hot-air drying requires a long time, which can cause a loss in nutritional quality and the degradation of bioactive compounds. However, the use of acidulants and reducing agents such as citric acid can minimize the activity of polyphenol oxidase enzyme (PPO) [28] and browning by lowering the pH of the product. To the best of our knowledge, no study has so far investigated the joint effect of oven drying and citric acid on the phenolic content of artichoke by-products powder.

This study explores the effect of the inclusion of artichoke outer bract powder on the bioactive compound content of gluten-free rusks, in terms of undigestible carbohydrates and phenolic compound content. The preparation of the artichoke powder as a functional ingredient was also optimized by considering two key factors: drying time and pre-treatment of artichoke bracts with food-grade citric acid. Two different composite gluten-free flours were tested, and the nutritional quality of the enriched rusks was determined.

## 2. Materials and Methods

### 2.1. Plant Material and Chemicals

Artichoke heads, “Mazzaferrata” ecotype, were harvested in situ at their place of origin (Abruzzo, Central Italy) and shipped to the laboratory of CREA Research Centre for Food and Nutrition (Rome, Italy).

The following chemicals were purchased from Carlo Erba Reagents (Milan, Italy): aluminum chloride, bovine serum albumin, calcium chloride, calcium chloride dihydrate, citric acid, disodium phosphate anhydrate, disodium phosphate monobasic monohydrate, ethanol, Folin–Ciocalteu reagent (FCR), glacial acetic acid, maleic acid, hydrochloric acid, *p*-hydroxybenzoic acid hydrazide, RPE methanol, sodium acetate, sodium borohydrade, sodium hydroxide, sodium maleate, sodium nitrate, sodium hydroxide, trysodium citrate dihydrate. Megazyme^®^ Fructan and Total Dietary Fiber kits were purchased from Astori Tecnica (Poncarale, Italy).

### 2.2. Upcycling of Artichoke Outer Bracts and Preparation of Powders

**Figure 1:**
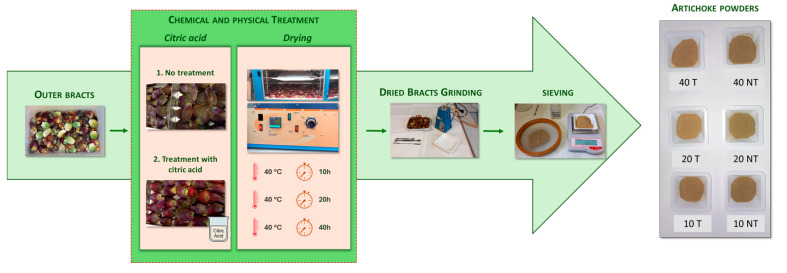
Upcycling the Outer Bracts of Artichokes for Value-Added Applications (T: treated with citric acid; NT: not treated with citric acid).

Upon arrival, outer bracts were removed from the artichoke head (52% of the total weight of the head) and washed with a solution of distilled water and sodium hypochlorite (1.25%) (Figure 1).

The drying protocol was optimized based on two key factors: (i) treatment of artichoke bracts with citric acid and (ii) drying time. An aliquot was treated with citric acid (15% *w*/*v* of solution) to evaluate the effect of bract pre-treatment on artichoke powder quality, while no treatment was performed on the remaining bracts. The aliquots were transferred into a ventilation oven (Intercontinental srl, Roma, Italy), and drying time was performed at atmospheric pressure at 40 °C for 10, 20, and 40 h. At pre-set intervals, samples were removed from the oven and weighed immediately after. The weights and relative times were noted in an Excel spreadsheet to obtain a drying curve.

Dried bracts were finely ground with a laboratory mill (Janke and Kunkel IKA Labortechnik, Staufen, Germany) equipped with a water-cooling system. The powder obtained was sieved (mesh size 670 µm). The six artichoke bract powder samples were stored in a desiccator until analysis. The drying condition tests were performed in duplicate.

### 2.3. Preparation of Gluten-Free Rusks

**Figure 2:**
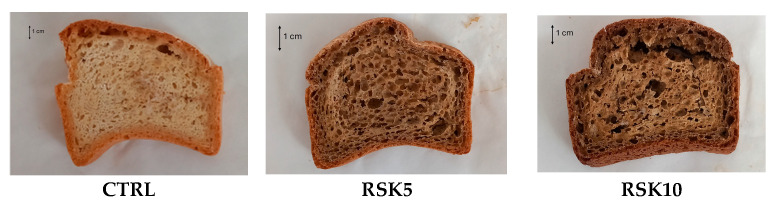
Experimental GF rusks (CTRL: GF rusks; RSK5: GF rusks with 5% artichoke powder; RSK10: GF rusks with 10% artichoke powder).

Three experimental GF rusk samples were prepared: one control and two fortified samples. The basic formulation of the control (CTRL) was as follows: commercial GF flour, baker yeast (2% GF flour weight), vegetable oil (6% flour weight), salt (1% GF flour weight), and water. The amount of water was determined following farinographic analysis. Enriched rusks were prepared by partially replacing the GF flour with artichoke outer bract powder. Two substitution levels were tested: 5% (RSK5) and 10% (RSK10). The doughs were prepared by kneading the ingredients in a mixer (Sottoriva, Marano—Vicenza, Italy) for 10 min. The bulk dough was proofed in a climate chamber for 30 min at 30 °C at 80% RH. Then, it was divided into three parts and placed in pans to obtain three loaves. Each loaf was proofed for 25 min and baked by an electric oven (Forno Angelo Po G20) for about 35 min at 200 °C and 30% HR. After baking, the loaves were placed at 30 °C for 24 h to promote the migration of water from the core of the loaf to the surface. Then, they were cut into 1.5 cm thick slices and dried at 140 °C for 20 min (Figure 2). Two batches for each experimental sample were prepared.

### 2.4. Selection of Artichoke Outer Bract Powders for GF Rusk Enrichment

Artichoke outer bract powders were analyzed for color, inulin, and free phenolic compound content to identify the powder with the highest values of color indices L*, a*, and b*, and the highest inulin and free phenolic compound content.

#### 2.4.1. Measurement of Color by the CIEL*a*b* System

The color of the artichoke powder was measured according to the CIELAB color system. The lightness (L*), red (a*), and yellow (b*) colorimetric indices were determined and acquired by a Chroma Meter CR 300 Minolta (Konica Minolta, Inc., Tokyo, Japan) equipped with a pulsed xenon lamp and illuminant D65. A white plate (X ¼ 91.98, Y ¼ 93.97, Z ¼ 110.41) was used to calibrate the instrument. The powder was pressed in a cell holder to achieve a consistent sample, and color values were read at five different points on the glass surface to give the average values. Results were expressed as mean values of independent measurements ± standard deviation.

The total color difference (ΔE) for the powders was also calculated using the following equation:

(1) $$\Delta E=\sqrt{{\Delta L}^{2}+{\Delta a}^{2}+{\Delta b}^{2}}$$

#### 2.4.2. Determination of Inulin Content in Artichoke Powders

Inulin content was determined by the Megazyme^®^ Fructan Assay kit, according to the manufacturer’s instructions and in accordance with AOAC Official Method No. 999.03 [29]. Briefly, the fructan assay procedure consisted of four steps: (i) fructan extraction in a boiling water bath; (ii) removal of sucrose, starch, and reducing sugars; (iii) hydrolysis and measurement of fructan; (iv) calculation. Results were expressed as % *w*/*w*. Fructans were determined in triplicate.

#### 2.4.3. Determination of Free Phenolic Compound Content in Artichoke Powders

A two-step extraction was carried out by coupling traditional solid–liquid extraction and ultrasound-assisted extraction (UAE), as reported in Melini et al. [10,30,31]. Briefly, a known amount of artichoke bract powder was placed into a PYREX™ screw cap culture tube, added with methanol: water (80:20 *v*/*v*), and vortexed to ensure mixing. The tube was then transferred in an ultrasound bath system (Elmasonic S 100 H, Elma Schmidbauer GmbH, Germany), operating at 37 kHz, and allowed for acclimatation to the water bath temperature for 2 min. UAE was performed at 60 °C for a total of 20 min, as reported in Melini et al. [10]. Ultrasound water bath temperature was monitored to keep the temperature constant.

After the first extraction, the solid–liquid solution was refrigerated at a temperature of +4 °C for 5 min; then, it was centrifuged (RS-10M, Remi Elektrotechnik Ltd., Mumbai, India; 7000 rpm, 10 min), and, finally, the supernatant was collected and stored for analysis. A second extraction was performed according to the same procedure and under the same conditions. The two supernatants were finally combined, and phenolic compounds were determined in the pooled phenolic extracts.

Free phenolic compounds (FPCs) were determined using the FCR assay, as reported by Sompong et al. [32] and Melini et al. [10,30,31]. Briefly, an aliquot of filtered phenolic extract was added to water-diluted FCR (1:10). Then, sodium carbonate (75 g/L) was added. Test tubes were transferred in a water bath (50 °C, 10 min). After cooling in the dark, the absorbance was measured at a wavelength of 760 nm against the blank reagent. For each extract, three replicates (*n* = 3) were performed.

FPCs were quantified using a calibration curve of pure gallic acid within a 22–121 µg mL^−1^ concentration range. Data were expressed as milligrams of gallic acid equivalents per 100 g of sample dry matter (mg GAE 100 g^−1^ dm).

### 2.5. Nutritional Characterization of the Artichoke Bract Powder Selected for Enrichment and Gluten-Free Rusks

Proximate composition, non-digestible carbohydrate content, free phenolic compound content and free flavonoid content were determined in the artichoke bract powder obtained under the optimized drying conditions, as well as in gluten-free rusks.

#### 2.5.1. Proximate Analysis

Moisture content was determined according to the AOAC Official Method No. 935.25 [29]. Protein content was determined based on total nitrogen content (N × 6.25), according to the ICC standard method No. 105/2 [33]. Crude fat content was assessed according to the AOAC Official Method No. 920.85 [29]. Ash content was evaluated according to the AOAC Official Method No. 923.03 [29]. Total carbohydrate content was obtained as a difference. Results were expressed as g/100 g dm.

#### 2.5.2. Analysis of Non-Digestible Carbohydrates 

##### Determination of Inulin Content

Inulin content was determined as reported in Section 2.4.2.

##### Determination of Total Dietary Fiber

Total dietary fiber (TDF) content was determined on dried and low-fat samples by the Megazyme^®^ Total Dietary Fiber Assay kit, according to the manufacturer’s instructions and in accordance with AOAC Official Method No. 985.29 [29]. Briefly, the sample was first subjected to sequential enzymatic digestion by heat-stable α-amylase, protease, and amyloglucosidase. Dietary fiber was precipitated with ethanol, filtered, and dried. Residues were corrected for protein, ash, and blank, and TDF content was calculated. TDF was determined in triplicate.

#### 2.5.3. Determination of Free Phenolic Compound Content

Free phenolic compounds were extracted as reported in Section 2.4.3. and determined spectrophotometrically as specified in Section 2.4.3.

Data were expressed as milligrams of gallic acid equivalents per 100 g of sample dry matter (mg GAE 100 g^−1^ dm).

#### 2.5.4. Determination of Total Flavonoid Compound Content

The content of Total Flavonoid Compounds (TFCs) was determined with a colorimetric assay, according to the procedure reported by Alshikh et al. [34], with slight modifications. Briefly, an aliquot of the phenolic extract obtained, as reported in Section 2.4.3., was mixed with distilled water and sodium nitrate 5% (*w*/*v*). The test tube was allowed to stand for 5 min until the reaction was complete, and then aluminum chloride 10% (*w*/*v*) was added to the mixture. Finally, sodium hydroxide 1 M and distilled water were added and mixed. Tubes were left in the dark at room temperature for 15 min. Absorbance was measured at a wavelength of 510 nm against the blank reagent. For each extract, three replicates (*n* = 3) were performed. A calibration curve of catechin, with a concentration ranging from 2 to 20 µg mL^−1^, was used to quantify total flavonoids. Results were expressed as milligrams of catechin equivalents (CE) per 100 g of sample on a dry matter basis (mg CE 100 g^−1^ dm).

### 2.6. Statistical Analysis

Statistical analysis was performed with the software Minitab Pro 18 (Minitab Inc., State College, PA, USA). Microsoft^®^ Excel^®^ for Windows 365 (version 2103) was also used to process the experiment data. Differences were tested by ANOVA followed by Tukey’s test. Statistically significant differences were reported for *p* < 0.05.

## 3. Results and Discussion

### 3.1. Drying Curve

**Figure 3:**
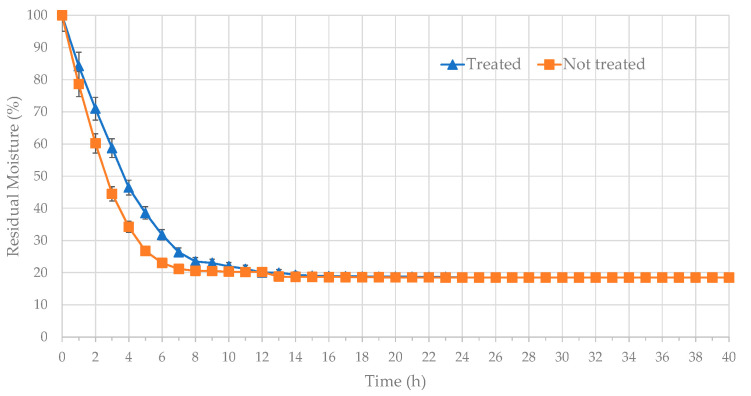
Kinetics of the residual moisture of artichoke outer bracts (■ not treated with citric acid, ▲ treated with citric acid).

The drying curve of the artichoke external bracts, either treated or untreated with citric acid, is shown in Figure 3. A temperature of 40 °C was selected, as it enables better preservation of plant tissue color during the drying process [35]. Moisture loss was measured at regular intervals till constant weight. After a 10-h drying process at 40 °C, the relative moisture content of the samples treated with citric acid was 22.0%, while the untreated samples showed a moisture content of 20.3% (Figure 3). After 20 h of drying, the relative moisture content was 18.7% and 18.5% in treated and untreated bracts, respectively. From 20 h to 40 h, the percentage of weight variation was less than 2% in both treated and untreated samples over three consecutive measurements. Hence, a drying time of 20 h is sufficient to achieve equilibrium moisture content.

To the best of our knowledge, there is a lack of investigations into the drying protocols that best preserve bioactive constituents during the conversion of artichoke by-products into powder form. Drying at 40 °C is generally performed for longer than 20 h. Canale et al. dried stems and bracts at 40 °C for about 48 h by using a forced convection drying chamber [36]; Boubaker et al. dried stems at 40 °C for 72 h [37] and bracts for 3 days [26]; Mejri et al. dried floral stems at 40 °C for one week [27]. Ruiz-Cano et al. dried bracts and stems for 24 h but applied a higher drying temperature (70 °C) [38]. Nevertheless, prolonged drying times or elevated temperatures may cause excessive damage to plant tissues and negatively affect the antioxidant compound content [39] and inulin levels [40].

### 3.2. Effect of Pre-Drying Treatment and Drying Conditions on Artichoke Bract Powders

Color is one of the key attributes affecting consumer perceptions and acceptability of food; therefore, the management of this factor is of paramount importance [41]. Bakery products formulated with the addition of plant-based powders are commonly less appealing because of their darker and greener notes compared to their cereal-based counterparts [42,43,44]. Hence, obtaining powders from vegetable by-products with high L*, a*, and b* values is crucial.

In this study, the effect of both treatment with citric acid and drying times on the color profile of artichoke outer bract powders was evaluated. The L* (lightness), a* (red/green coordinate), and b* (yellow/blue coordinate) values are reported in Table 1. Citric acid treatment increased the brightness (L*), redness (a*), and yellowness (b*) of the artichoke powders. The highest L* and a* values were obtained in samples treated with citric acid and dried for 20 and 40 h. The a* index values were positive in all samples. In contrast, previous studies by Boubaker et al. [37], Canale et al. [36], Cannas et al. [17], and Umaña et al. [45] reported negative a*values for artichoke stem and bract flour. This discrepancy may be attributed to genetic differences and variations in drying conditions, which likely influenced powder color. Moreover, in this study, the treatment with citric acid helped preserve the violet color in bracts, contributing to the reddish shades observed in the final powder. As regards yellowness, the highest b* values were obtained when artichoke bracts were dried for 10 and 20 h; hence, these powders might contribute maintaining a yellow hue in bakery products, which is appreciated by consumers. Based on the L*, a*, and b* values, the total color difference (ΔE) of artichoke powders, compared to GF control flour, was calculated. It emerged that the powder treated with citric acid and dried for 20 and 40 h had the lowest color difference (ΔE) values (Table 1).

Based on the experimental data of this study, it can be stated that using powders obtained from bracts treated with citric acid and dried for a maximum of 20 h allows the production of food products that meet consumer expectations in terms of color.

Currently, most inulin used for nutraceutical applications is derived from Jerusalem artichoke (*Helianthus tuberosus* L.) and chicory (*Cichorium intybus* L.) [46]. Nonetheless, globe artichoke also represents a valuable inulin source [46]; thus, its use as a functional ingredient in food (e.g., bakery, extruded, and dairy products) is worth being explored.

The effect of drying conditions on the inulin content was investigated in the six artichoke powders. Results showed that the treatment with citric acid reduced inulin content (Table 1), likely due to acid-induced breakdown of inulin [40]. Moreover, bracts dried for longer time (40 h) exhibited a lower inulin content, in keeping with the data reviewed by Kheto et al. [40]. No significant differences were observed between bracts dried for 10 and 20 h.

Free phenolic compounds in artichoke outer bract powders are reported in Table 1. Drying time was found to significantly affect FPC content. The highest value was observed in samples dried for 20 h. FPC content was 783.49 mg GAE/100 g in bracts treated with citric acid, compared to 727.49 mg GAE/100 g in untreated samples. These values were higher than those in samples dried for 10 h, because thermal treatments can promote the accessibility and release bioactive compounds from the food matrix [47]. Samples dried for 40 h showed lower FPC content values than those dried for 20 h. This might be due to thermal degradation of antioxidant compounds [47]. As regards the effect of citric acid treatment on FPC content, no significant differences were observed (Table 1).

Based on the data obtained for color profile, inulin, and free phenolic compound content, the powder produced by treating outer bracts with citric acid and drying for 20 h was selected for the enrichment of GF rusks.

### 3.3. Proximate Composition and Bioactive Compounds in the Selected Artichoke Bract Powder, in Gluten-Free Flours, and in Enriched Rusks

The artichoke bract powder produced by citric acid treatment followed by 20 h of drying, as well as the GF blends and experimental rusks, were analyzed for proximate composition, non-digestible carbohydrates, and phenolic and flavonoid compound content.

#### 3.3.1. Proximate Composition

The proximate composition of (i) the artichoke bract powder obtained by 20 h drying after treatment with citric acid, (ii) GF flour blends, and (iii) experimental GF rusks is reported in Table 2.

The moisture content of the artichoke powder was 11.62%, which is higher than values reported in the literature for powders obtained from artichoke by-products. Cannas et al. found moisture contents of 5.61 and 6.12 g/100 g in powders obtained from freeze-dried bracts and stems, respectively [17]. Similarly, Canale et al. reported a moisture content of 4 g/100 g in flours obtained from artichoke bracts dried at 40 ± 5 °C for 48 h in a forced convection drying chamber [36]. However, the powder moisture content found in the present study (Table 2) is close to typical values reported for wheat and whole meal flours (13–14%) [48]. Considering the intended use of the powder as an ingredient in the formulation of other foods, moisture content is a critical quality parameter. Moisture levels affect, in fact, the physical and chemical stability of food powders. Excess moisture can lead to spoilage, reduced shelf-life, and compromised structural integrity, while excessively low moisture levels can negatively impact powder binding. Moreover, moisture content influences the flowability of bulk solids, which can affect the efficiency of manufacturing processes [49]. In the GF flours used in this study, moisture content ranged between 10.56 ± 0.02 g/100 g and 11.03 ± 0.16 g/100 g. In the rusks, moisture levels ranged from 7.95 ± 0.08 to 8.49 ± 0.06, with RSK10 showing the highest moisture content. These results are consistent with those reported by Devpal et al. [14].

As regards protein content, the artichoke bract powder obtained in this study was rich in protein (15.43 ± 0.02 g/100 g dm) (Table 2). This value is in keeping with the data reported by Umaña et al., who found approximately 14.88 ± 0.93 g/100 g in powders obtained from freeze-dried bracts from artichokes grown in Spain [45]. It is also comparable to the protein content reported by Ruiz-Cano et al. [38] for bracts of the Spanish cultivar ”Blanca de Tudela” dried at 70 °C for 24 h (10.5–15.2 g/100 g dm). On the other hand, the protein content in this study was higher than that reported by Boubaker et al. for stems from the “Violet d’Hyères” artichoke variety, dried at 40 °C for 72 h (6.66 ± 0.52 g/100 g dm) [37]; by Cannas et al. for freeze-dried outer bracts of the Italian PDO cultivar “Carciofo Spinoso di Sardegna” (9.48 ± 0.18 g/100 g dm) [17]; and by Boubaker et al. for a Tunisian cultivar of artichoke stem powder (11.53 g/100 g dm) [26]. This variability can likely be attributed to genetic differences among artichoke cultivars, as protein content is known to be variety-dependent [50,51]. The incorporation of proteins from different sources can influence the nutritional, chemical, physical, and functional properties of the final product [52]. The powder protein content of the artichoke bract powder in this study is comparable to that of whole meal flour (10–15%) and wheat flour (8–13%) [48]. Therefore, the artichoke by-product powder shows promise for partial replacement of wheat flour in functional bakery products and/or pasta formulations. The substitution of GF flours with 5% and 10% artichoke outer bract powder significantly (*p* < 0.05) increased the protein content (Table 2). In the GF rusks, protein content ranged between 3.98 ± 0.02 and 5.30 ± 0.05 g/100 g dm, with both RSK5 and RSK10 being richer in protein than the control GF rusks. Specifically, substitution with 5% and 10% artichoke outer bract powder increased protein content by 19% and 33%, respectively, with the highest value recorded in F-RSK10 (Table 2). Similar trends were observed in the flour blends, where F-RSK10 had the highest protein content. When comparing the experimental rusks with commercially available GF ones, the inclusion of artichoke powder was shown to effectively produce a protein-enriched product.

The fat content of the artichoke powder obtained from dried external bracts was approximately 0.77 g/100 g dm (Table 2). This value is lower than that reported by Umaña et al. for freeze-dried artichoke bracts (1.24 g/100 g) [45], but in keeping with the values recorded for artichoke by-products in other studies [17,26]. The experimental GF flours, produced by partially substituting the control flour with 5% and 10% artichoke outer bract powder, showed no significant differences in fat content compared to the control flour (*p* > 0.05). In the rusks, fat content ranged from 3.36 ± 0.06 to 3.61 ± 0.04 g/100 g dm, with no significant differences observed (*p* < 0.05). This can be attributed to the low-fat content of the artichoke powder. Furthermore, the experimental rusks exhibited lower fat levels compared to commercial GF rusks, which typically contain 5.4–6.4 g fat/100 g, as indicated on product labels.

The ash content of the artichoke powder (7.22 ± 0.12 g/100 g dm; Table 2) was consistent with values reported in the literature, such as 6.16 ± 0.01 and 7.19 ± 0.04 g/100 g dm for powders obtained from bracts and stems, respectively [17]. The inclusion of artichoke powder in GF flour significantly (*p* < 0.05) increased ash content (Table 2). In the GF rusks, ash content was lowest in the control sample and highest in RSK10 (Table 2), indicating that the enrichment significantly affected ash content.

In the context of using the powder obtained in this study for the enrichment of bakery products or pasta, its high protein and low-fat content suggest that it could be a valuable ingredient, particularly for the formulation of foods intended for special dietary needs [13]. The protein content of the artichoke bract powder is higher than that of other ingredients commonly used in the formulation of gluten-free foods, such as rice flour (8.52 g/100 g dm) [53] and corn flour (6.43 g/100 g) [54]. Moreover, it is comparable to raw materials that are increasingly explored for their nutritional potential as plant-based protein sources, including quinoa flour (protein content: 15.7 g/100 g dm) [55], buckwheat flour (protein content: 13.07 g/100 g dm) [56], and chickpea flour (protein content: 17–22 g/100 g dm) [57].

#### 3.3.2. Non-Digestible Carbohydrates

Total dietary fiber and inulin content were determined in the powder obtained from artichoke external bracts under optimized processing conditions, in the GF flour control and blends thereof, as well as in the experimental rusks.

The total dietary fiber content of the artichoke powder was 39.69 g/100 g dm (Table 2). This value is lower than those reported by Quintero-Ruiz et al. for non-edible artichoke bracts from Argentine and Italian artichoke cultivars (55.25–60.01 g/100 g dm) [22], and lower than the value found by Boubaker et al. for artichoke stem powder (51.29 g/100 g dm) [26]. It is lower than the value reported by Umaña et al. for artichoke bract flour (66.59 g/100 g dm) [45]. However, the fiber content obtained in the present study was higher than that reported by Cannas et al. for freeze-dried bracts and stems (68.57 and 43.63 mg/100 g dm, respectively) [17].

In the GF control flour and its blends, an increase in TDF was observed following the incorporation of artichoke by-product powder. In F-RSK10, TDF was almost three-fold higher than in F-CTRL sample. In the experimental GF rusks, TDF was 4.51 ± 0.59 g/100 g in RSK5 and 7.67 ± 0.72 g/100 g in RSK10 (Table 2). Thus, the enrichment resulted in a higher fiber content, compared to the standard GF rusks. Consequently, the consumption of the enriched rusks contributes to improving dietary fiber intake in coeliac subjects. Additionally, RSK10 meets the nutritional criteria for the claim “high fiber”. The dietary fiber content RSK10 is comparable to that reported for some commercial GF rusks, which typically contain around 8 g/100 g.

The inulin content of the artichoke outer bract powder was 6.41 g/100 g dm (Table 2), a value that is consistent with that reported by Canale et al., who found 6.5 g/100 g inulin in bracts from artichokes of the “Violetto ramacchese” cultivar, dried at 40 ± 5 °C for about 48 h using a forced convection drying chamber [36]. A higher inulin content (15.05 g/100 g dm) was reported for artichoke bracts flour [45]. Blending the F-CTRL flour with artichoke by-product powder did not significantly affect inulin content when the inclusion level was 5%, whereas a significant increase was observed in F-RSK10 (Table 2). In the experimental GF rusks, inulin content was 0.33 ± 0.05 g/100 g dm in RSK5 and 0.83 ± 0.07 g/100 g dm in RSK10. This reflects a two- and four-fold increase, respectively, compared to the control. Since inulin is widely used as a fat replacer, future research could explore reducing the percentage of vegetable oil in GF rusk formulations to lower overall fat content. This would be particularly valuable from a nutritional perspective, as GF products are often characterized by high fat levels [15]. The decrease in inulin content observed in the GF rusks, compared to the corresponding GF flour blends, is likely attributable to fermentation; during leavening, fructans are subject to degradation by invertase enzymes released by *Saccharomyces cerevisiae* [58].

The results obtained in this study confirm that the addition of artichoke powder represents a promising innovation strategy for the development of food products with enhanced nutritional value. Dietary fiber and inulin, both classified non-digestible carbohydrates (NDCs), are well-recognized for their physiological benefits. Scientific evidence has demonstrated their positive effect on human health, including reduction in blood glucose, cholesterol, and blood pressure, improvement of laxation, and increased intestinal mineral absorption [59]. In particular, the incorporation of artichoke powder into food formulations (e.g., bakery products, pasta, etc.), expands the range of products available for special dietary regimes requiring fiber supplementation.

At the same time, enriching food products with plant-based powders presents some technological challenges, primarily due to the intrinsic properties of dietary fiber, particularly its water-holding capacity. This characteristic can lead to competitive interactions between the added fiber and wheat proteins, hindering the gluten network formation and consequently resulting in denser and firmer baked products [60]. On the other hand, increased water retention can be advantageous from both an anti-staling perspective and in the production of gluten-free bakery products, where the absence of gluten makes water-binding components desirable for improving texture and shelf life [60].

#### 3.3.3. Phenolic Compounds

Artichoke outer bracts are also a rich source of phytochemicals, including phenolic compounds, which contribute to the antioxidant capacity and potential health-promoting properties of the food matrix.

The phenolic content of the artichoke bract powder obtained in this study was 708.65 ± 33.47 mg GAE 100 g^−1^ dm, (Table 2). Data on the content of free phenolic compounds in artichoke by-products are available in the literature [17,19,20,21,24,27,38,45,61,62]; however, it is important to note that no official methods currently exist for the quantification of these compounds. Therefore, when comparing data on phenolic compound content across studies, external factors, such as the solvent used (e.g., methanol, ethanol), the extraction technology (e.g., microwave-assisted, ultrasound-assisted, and pressurized liquid extraction) and the specific extraction conditions (e.g., solvent concentration, time, temperature), must be considered [10,30].

Replacing GF flour with artichoke powder significantly (*p* < 0.05) increased the content of free phenolic compounds. The higher the level of substitution, the higher the FPC observed. In the experimental GF rusks, FPC was 56.35 ± 1.25 mg GAE 100 g^−1^ dm in the control and it increased by 1.7 and2.5-fold with 5% and 10% artichoke powder substitution, respectively.

In the literature, a content of about 1350 ± 0.07 mg GAE/100 g dm was reported for dried artichoke stems extracted with ethanol (80%) by magnetic stirring for 24 h at 37 °C. Free phenolic content ranged from 2.2 to 0.8 mg GAE/100 g dm and 4 to 9 mg GAE/100 g dm in bracts and stems, respectively, dried at different temperatures (40 to 120 °C) [62]. Discarded by-products of the “Spinoso sardo” globe artichoke showed high free phenolic compound levels, depending on the extraction technique used (maceration and ultrasound-assisted extraction). It ranged from 1865.56 ± 4.93 to 2014.40 mg GAE/100 g dm in bracts, 1723.10 ± 20.03 to 1863.26 ± 5.81 in leaves, and 2516.03 ± 4.35 to 2603.50 ± 10.33 in stems [63]. In another study on the upcycling of two “Spinoso sardo” globe artichoke by-products (stems and bracts), FPC was significantly lower in bracts (951.52 ± 2.01 mg GAE/100 g dm) compared to stems (1258.87 ± 7.58 mg GAE/100 g dm) [17]. Colantuomo et al. also reported high FPC in artichoke leaves and stems (2160 and 3470 mg GAE/100 g dm, respectively), with extraction performed using ultrasounds and acidified methanol as a solvent [61]. Additionally, FPC values ranging from 63.33 to 80.83 mg GAE/g extract were found in inedible floral stems oven-dried at 40 °C for one week and extracted using various organic solvents, such as butanol, ethyl acetate, ethanol, and methanol, via maceration [27].

As regards flavonoid content, the artichoke outer bract powder contained 5903.06 ± 42.38 mg CE 100 g^−1^ dm (Table 2). This value is higher than the flavonoid content previously reported for “Spinoso sardo” globe artichoke by-products, where concentrations were 564.31 mg CE/100 g dm in bracts and 1233.9 ± 18.71 mg CE/100 g dm in stems [17]. The substitution of GF flour with artichoke powder significantly (*p* < 0.05) increased TFC (Table 2). In the experimental rusks, TFC ranged between 221.69 ± 9.48 and 123.43 ± 32.78 mg CE 100 g^−1^ dm. Incorporation of 5% and 10% artichoke outer bract powder led to a 4- to 5-fold increase in flavonoid content compared to the control, respectively.

### 3.4. Limitations and Future Perspectives of the Study

Food reformulation aims at either reducing ingredients or nutrients of concern, such as salt, added sugar, and saturated and trans fats, or to increase the content of health-promoting components, such as dietary fiber and antioxidants. Food reformulation contributes to shifting towards healthier and more sustainable consumption patterns. However, it often poses challenges related to the sensory characteristics of food products, including appearance, texture and flavor. In this study, the effect of artichoke outer bract powder on the nutritional profile and color of gluten-free rusks was evaluated. Color plays a crucial role in consumer food choice, influencing not only acceptability and preference, but also taste thresholds and sweetness perception.

Since sensory evaluation combined with texture analysis provides essential information on product attributes and overall liking, further studies are planned. They help in fact to better direct food waste processing and food formulation, as a next step to assess the acceptability of the GF rusks formulated in this study. These investigations will provide key insights to fine-tune both ingredient incorporation levels and processing conditions, ultimately facilitating the upcycling of vegetable by-products into value-added, consumer-accepted functional foods.

## 4. Conclusions

The artichoke bract powder produced in this study was characterized by high protein and fiber content, low fat levels, and a substantial concentration of bioactive compounds, such as free phenolics and flavonoids. These features make it a valuable ingredient, especially for the formulation of foods intended for special dietary needs, such as those of individuals with celiac disease.

The valorization of artichoke outer bracts as a functional ingredient offers a promising approach for enhancing the nutritional quality of gluten-free (GF) bakery products. Nutritionally, the inclusion of artichoke powder significantly increased the protein, ash, total dietary fiber, inulin, and bioactive compound content of the GF flour blends and rusks. In terms of bioactive compounds, the enrichment with artichoke powder substantially improved the antioxidant potential of the GF rusks, as indicated by increased free phenolic and flavonoid contents.

In conclusion, the use of artichoke outer bract powder represents a sustainable and innovative strategy for improving the nutritional and functional properties of gluten-free bakery products, contributing both to food waste reduction and to the development of products tailored for special dietary needs.

## Figures and Tables

**Table 1:** Color indexes and free phenolic compound content in artichoke powder samples.

Sample ID	Pretreatment	Drying Time (h)	L*	a*	b*	ΔE	Inulin (%)	FPCs (mg GAE/100 g dm)
10 T	Citric acid	10	59.00 ± 0.21 ^b,E^	1.82 ± 0.10 ^b,E^	21.83 ± 0.20 ^a,E^	38.02 ± 0.18 ^a^	18.90 ± 0.51 ^c,B^	336.50 ± 18.30 ^c,B^
10 NT	-	10	58.27 ± 0.42 ^c,d,F^	0.18 ± 0.16 ^e,F^	21.19 ± 0.14 ^b,F^	38.32 ± 0.36 ^a^	22.03 ± 0.16 ^a,A^	331.80 ± 14.40 ^c,B^
20 T	Citric acid	20	61.81 ± 0.26 ^a,A^	2.93 ± 0.09 ^a,A^	21.74 ± 0.17 ^a,A^	35.58 ± 0.30 ^c^	18.69 ± 0.07 ^c,D^	783.50 ± 10.10 ^a,A^
20 NT	-	20	57.74 ± 0.44 ^d,B^	0.73 ± 0.22 ^d,B^	20.89 ± 0.29 ^b,B^	38.73 ± 0.51 ^a^	21.73 ± 0.16 ^a,C^	777.50 ± 1.10 ^a,A^
40 T	Citric acid	40	61.73 ± 0.27 ^a,C^	2.83 ± 0.11 ^a,C^	21.23 ± 0.18 ^b,C^	35.43 ± 0.27 ^c^	17.35 ± 0.01 ^d,F^	481.30 ± 63.00 ^b,C^
40 NT	-	40	58.98 ± 0.37 ^b,c,D^	1.18 ± 0.05 ^c,D^	19.99 ± 0.21 ^c,D^	37.27 ± 0.30 ^b^	20.49 ± 0.01 ^b,E^	481.50 ± 83.80 ^b,C^

T = treated with citric acid; NT = not treated with citric acid; FPCs: free phenolic compounds. Data are presented as mean ± standard deviation (*n* = 3) and expressed on a dry matter basis (dm). Mean values with different small case letters within columns are significantly different based on *p* < 0.05 by analysis of variance followed by Tukey’s test. Upper-case letters refer to pairs (T vs. NT); ΔE was calculated with respect to GF control flour; mean values with different upper-case letters are significantly different (*p* < 0.05).

**Table 2:** Proximate composition, total dietary fibre, inulin and phenolic compound content in artichoke outer bract powder, GF flour blends and experimental rusks.

**Analyte**	**Artichoke** **Powder**	**GF Flours**			**Experimental Rusks**		
		**F-CTRL**	**F-RSK5**	**F-RSK10**	**CTRL**	**RSK5**	**RSK10**
Moisture (%)	11.62 ± 0.04	10.94 ± 0.06 ^A^	11.03 ± 0.16 ^A^	10.56 ± 0.02 ^B^	8.04 ± 0.13 ^b^	7.95 ± 0.08 ^b^	8.49 ± 0.06 ^a^
Protein (g/100 g dm)	15.43 ± 0.02	3.32 ± 0.05 ^C^	3.88 ± 0.06 ^B^	4.34 ± 0.03 ^A^	3.98 ± 0.02 ^c^	4.72 ± 0.04 ^b^	5.30 ± 0.05 ^a^
Fat (g/100 g dm)	0.77 ± 0.08	0.61 ± 0.08 ^A^	0.58 ± 0.01 ^A^	0.23 ± 0.04 ^B^	3.61 ± 0.04 ^a^	3.44 ± 0.10 ^a^	3.36 ± 0.06 ^a^
Carbohydrates (g/100 g dm)	76.68 ± 0.15	94.91 ± 0.29 ^A^	94.12 ± 0.06 ^A^	93.77 ± 0.34 ^A^	90.24 ± 0.05 ^a^	89.40 ± 0.11 ^a^	88.69 ± 0.08 ^a^
Ash (g/100 g dm)	7.12 ± 0.12	1.16 ± 0.02 ^C^	1.42 ± 0.02 ^B^	1.66 ± 0.00 ^A^	2.17 ± 0.20 ^c^	2.44 ± 0.00 ^b^	2.65 ± 0.03 ^a^
Total Dietary Fiber (g/100 g dm)	39.69 ± 0.05	1.17 ± 0.02 ^C^	3.65 ± 0.10 ^B^	4.82 ± 0.09 ^A^	3.81 ± 0.25 ^c^	4.51 ± 0.59 ^b^	7.67 ± 0.72 ^a^
Inulin (g/100 g dm)	6.41 ± 0.11	0.57 ± 0.01 ^B^	0.54 ± 0.04 ^B^	0.79 ± 0.09 ^A^	0.21 ± 0.02 ^c^	0.33 ± 0.05 ^b^	0.83 ± 0.07 ^a^
Phenolic compounds							
FPCs (mg GAE 100 g^−1^ dm)	708.65 ± 33.47	39.05 ± 3.39 ^C^	64.88 ± 2.82 ^B^	95.02 ± 3.73 ^A^	56.35 ± 1.25 ^c^	94.87 ± 7.06 ^b^	138.99 ± 11.45 ^a^
TFCs (mg CE 100 g^−1^ dm)	5903.06 ± 42.38	153.62 ± 31.6 ^C^	547.43 ± 121.47 ^B^	772.65 ± 48.48 ^A^	221.69 ± 9.48 ^c^	802.46 ± 15.65 ^b^	1123.43 ± 32.78 ^a^

Different upper-case letters indicate significant (*p* < 0.05) differences (Tukey test) among GF flours. Different lower-case letters indicate significant (*p* < 0.05) differences (Tukey-test) among the experimental GF rusks.

## Data Availability

The original contributions presented in this study are included in the article. Further inquiries can be directed to the corresponding author.

## Abbreviations

The following abbreviations are used in this manuscript:

FCR	Folin–Ciocalteu Reagent
UAE	Ultrasound-Assisted Extraction
FPCs	Free Phenolic Compounds
TDF	Total Dietary Fiber
BPC	Bound Phenolic Compounds
GAEs	Gallic Acid Equivalents
TFCs	Total Flavonoid Compounds
NDCs	Non-Digestible Carbohydrates
